# Special Issue “Hox Genes in Development: New Paradigms”

**DOI:** 10.3390/jdb10030034

**Published:** 2022-08-18

**Authors:** Samir Merabet

**Affiliations:** IGFL, ENS-Lyon, CNRS, University Lyon I, 32/34 Av. Tony Garnier, 69007 Lyon, France; samir.merabet@ens-lyon.fr

In this Special Issue on “Hox genes in development: new paradigms”, we present a compilation of articles and reviews tackling various aspects of the Hox biology field.

Hox genes are fascinating developmental regulators that control cell fate in all tissues and at all stages in most animal embryos. Their important roles are maintained in adults, in particular, to control the state of stem cells in different lineages. Not only the rules governing their typical expression profile, but also their molecular mode of action, have fueled a number of investigations in recent decades. Due to this large amount of work, Hox genes are amongst the best studied developmental gene family and are at the basis of important paradigms in developmental biology. Here, we present a series of reviews and articles that update our current knowledge on the mechanisms regulating Hox genes expression and Hox protein activity. This series covers a large window of the numerous Hox molecular facets and provides substantial support for the reader to better understand how Hox genes could have deployed such an astonishing molecular diversity during development and evolution.

This Special Issue contains three research articles and six reviews.

Two research articles are dedicated to HOX DNA-binding preferences and describe the unexpected enrichment of low-affinity DNA-binding sites in vivo, with interesting speculation on HOX-HOX heterodimer activity [1] and the emergence of tissue-specific low-affinity binding sites during evolution [2]. The third research article is related to the description of Hox maternal transcripts deposited in the oocyte of the Annelida *Platynereis dumerlii*, which recalls previous observations in mammals and highlights new perspectives for studying the maternal function of Hox genes [3]. 

Reviews recapitulate the role of HOX proteins in different developmental and cancer contexts, or describe the various molecular strategies that control the Hox gene expression profiles along the anteroposterior axis of the embryo. One review updates the previously defined “micro-manager” role of Hox genes to emphasize their subtle and precise function during post-embryonic development in *Drosophila* [4]. Another review highlights the importance of post-translational modifications for controlling Hox function in both the nuclear and cytoplasmic compartments [5]. Two reviews describe how recent findings on the molecular mechanisms governing transcriptional regulation could apply to Hox gene expression. These mechanisms relate to the complex relationships existing between different enhancers, or the role of Topologically Associated Domains (TADs) and long non-coding RNAs within Hox genes clusters [6,7]. In addition to these biological aspects, an additional review proposes the application of the groundbreaking theory to complement the biophysical model of Hox gene collinearity during development and evolution [8]. Finally, one review discussed the combinatorial role of HOX and MEIS proteins in leukemia and solid tumors [9]. Together, these two reviews underline the value of considering HOX proteins and the HOX-MEIS partnerships from a therapeutic perspective in cancer.

I would like to thank the authors and reviewers whose contribution was crucial in guaranteeing the scientific quality of this Special Issue. I hope these articles will attract newcomers in the astonishing field of Hox gene biology, which will continue to stimulate original research and paradigms in future years. 
